# Study on the Anti-Poison Performance of Al–Y–P Master Alloy for Impurity Ca in Aluminum Alloys

**DOI:** 10.3390/ma10121356

**Published:** 2017-11-26

**Authors:** Min Zuo, Yu Dong, Degang Zhao, Yan Wang, Xinying Teng

**Affiliations:** Shandong Provincial Key Laboratory of Preparation and Measurement of Building Materials, School of Materials Science and Engineering, University of Jinan, Jinan 250022, China; dongyu970915@icloud.com (Y.D.); mse_zhaodg@ujn.edu.cn (D.Z.); mse_wangy@ujn.edu.cn (Y.W.); mse_tengxy@ujn.edu.cn (X.T.)

**Keywords:** Al–Si alloy, microstructure, phosphide, calcium-rich compound, multi-encapsulation structure

## Abstract

In this article, the anti-poison performance of novel Al–6Y–2P master alloy for impurity Ca in hypereutectic Al–Si alloys was investigated in detail. According to the microstructural analysis, it can be found that the primary Si and eutectic Si particles could be relatively modified and refined. In order to investigate the influence mechanism of Ca on the limited refinement performance of Al–6Y–2P master alloy, types of Al–xSi–2Ca–3Y–1P (*x* = 0, 6, 12, 18, and 30) alloys were prepared. It is observed that Ca takes the form of more stable Ca_3_P_2_ compounds by reacting with YP, and the surface of Ca_3_P_2_ particles are unsmooth, and even some have wrinkles in Al Al–2Ca–3Y–1P alloy. With the increase of Si content in Al–xSi–2Ca–3Y–1P (*x* = 6, 12, 18 and 30) systems, the multi-encapsulation structures, i.e., the phosphide (AlP and YP), hexagonal Al_2_Si_2_Ca, the Al_3_Si_2_Y_2_ or primary Si from inside to outside in order were examined.The excapsulation of YP and AlP caused by Al_2_Si_2_Ca might be the reason for the limited refinement effect of Al–6Y–2P master alloy for hypereutectic Al–18Si alloys.

## 1. Introduction

Nowadays, hypereutectic Al–Si alloys have been considered to be one of the ideal candidates for structural applications in automotive and aerospace industries due to their high specific strength, good castability, excellent wear and corrosion resistance, higher thermal stability and superior mechanical properties [1,2,3,4,5,6,7]. However, as the spots of crack initiation, the morphologies and distributions of both primary and eutectic Si particles have a significant influence on the mechanical properties of Al–Si alloys. Therefore, these precipitations must be effectively modified and refined to improve the mechanical properties of aluminum alloys.

Many researchers have focused on the modification and refinement of Si particles to obtain the optimal mechanical properties with wider applications. Various techniques have been improved to obtain the microstructure refinement of hypereutectic Al–Si alloys, such as spray forming [8,9,10], melt modification agent [11,12,13], melt thermal-rate treatment [14], equal channel angular pressing (ECAP) [15,16], squeeze casting [17], and so on. Among these techniques, chemical melt agent is well accepted as an excellent modifier of Si precipitation of aluminum alloy. By adding trace amount of phosphorus into the melt, the primary Si in hypereutectic Al–Si alloys can be significantly refined from about 100 μm to 30 μm, with the distribution improved. This was attributed to the presence of AlP, which could act as the ideal heterogeneous nucleation sites of Si phase due to the very similar lattice parameters [18]. Up to now, types of master alloys containing phosphorus have been designed to meet the requirement of industry applications, including Cu–P [19], Al–P [3,20], Al–Cu–P, Al–Fe–P [21], Al–Si–P [22], Al–Zr–P [23], Al–RE–P [24], and so on. Among these master alloys, novel Al–6Y–2P master alloy containing YP particles has drawn much attention for the excellent composite modification performance for hypereutectic Al–Si alloys. It was reported that the Si atoms in the melt could promote the structural evolution of YP to AlP with the release of Y, which just be reason for the excellent composite modification performance of developed Al–6Y–2P master alloy [24]. While, the influence of melt state on the modification performance of this novel master alloy needs further study in detail. 

In Al–Si alloys, Ca emerges as an element of concern, which is influenced by the grade of crystalline Si used as alloying element [25,26,27]. Ludwig et al. [27] reported that Ca element could suppress characteristic temperature that is associated with nucleation and the growth of the eutectic structure. Furthermore, it was found that Ca could significantly modify the eutectic Si and precipitate in the form of polyhedral Al_2_Si_2_Ca phase with the concentration as low as 39 ppm. Zaldívar-Cadena and Flore-Valdés [28] observed that the calcium-rich particles (hexagonal Al_2_Si_2_Ca) present needle-like or plate-like morphologies. Liu et al. [29] studied calcium-rich compounds in near eutectic Al–Si alloys, and reported that Ca could lead to phosphorus modification inefficiency due to the formation of Ca_x_Si_y_P_z_ compounds. 

The trace element Ca in raw materials is inevitable, which has a harmful influence on the modification and refinement effect of master alloys containing phosphorus. In this study, novel Al–6Y–2P master alloy with YP particles was used to carry out the melt treatment of hypereutectic Al–18Si alloys, and the influence of Ca on the composite modification of this master alloy was studied in detail. Furthermore, the inhibition mechanism of Ca for this master alloy was also discussed.

## 2. Experimental Procedures

The commercial purity of rare earth element yttrium and binary Al–3.5P master alloys were applied to synthesize novel Al–6Y–2P master alloy in vacuum arc melting furnace (XH, Beijing Institute of Huiguang, Beijing, China) under argon atmosphere (all of the compositions quoted in this work are in wt.% unless otherwise stated). In order to obtain the compositional homogeneity, this master alloy was remelted for three times. The binary Al–3.5P master alloys were provided by Shandong Al&Mg Melt Technology Co. Ltd. (Jinan, China). Element calcium was added into the Al–Si alloy in the form of Al–29Ca master alloy. Industrial pure aluminum (99.9%) and crystalline silicon (99.9%) were used to prepare the base Al–Si alloys.

The melt treatments of Al–18Si alloys were carried out as follows. The base alloy was remelted in a graphite crucible by using an electrical resistance furnace and then kept at the melting temperature for 30 min. The first two groups of melt treatment experiments were carried out with the addition of 0.2% Al–29Ca and 1.5% Al–6Y–2P master alloys, respectively. Then, both of the master alloys were added into the melt simultaneously to investigate the inhibition effect of Ca on the modification performance of Al–6Y–2P master alloy (third group). The melt temperature was controlled by the thermocouple and was assisted by the K-model handset thermocouple (K-model, Taizhou Hualongte Electric Co. Ltd., Taizhou, China) to make sure that the error was below ±5 °C. Finally, the melt was poured out into a permanent mold (70 × 35 × 20 mm^3^) preheated to 200 °C.

In order to investigate the relationship between the calcium-rich compounds and phosphides, types of Al–xSi–2Ca–3Y–1P (*x* = 0, 6, 12, 18 and 30) alloys were prepared using a medium frequency induction furnace. Metallographic specimens were all cut from the middle part of the casting samples, and then mechanically grounded and polished through standard routines. The average size of primary Si was measured from at least ten areas of the specimen metallograph, and in each area about ten primary Si particles were chosen. It is worth noting that the specimen of Al–xSi–2Ca–3Y–1P (*x* = 0, 6, 12, 18, and 30) alloys need to be fast prepared to avoid the oxidation and hydrolysis of phosphide compounds. The phase compositions were identified by X-ray diffraction (XRD) (D8 ADVANCE, Bruker, Germany) with Cu Kα operated with a voltage of 36 kv and an anode current of 26 mA. The characterizations of the microstructure and qualitative analysis were conducted by means of high scope video microscope (HSVM) (4XC, Shanghai Caikon Optical Instrument Co. LTD, Shanghai, China) and field emission scanning electron microscope (FESEM) (QUANTA FEG250, FEI, Hillsboro, OR, USA) equipped with energy dispersive X-ray spectrometer (EDS) (INCA Energy X-MAX-50 X, OIMS, UK). 

## 3. Results and Discussion

### 3.1. Typical Microstructures of Al–18Si Alloys with the Addition of Ca and/or P

**Figure 1:**
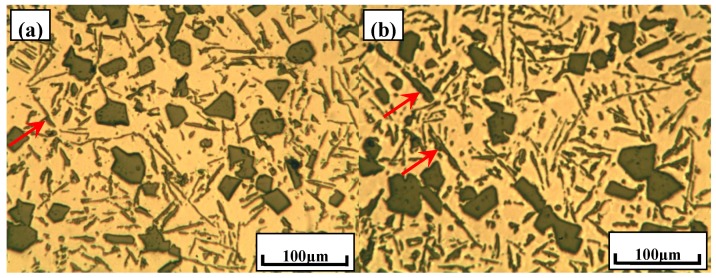

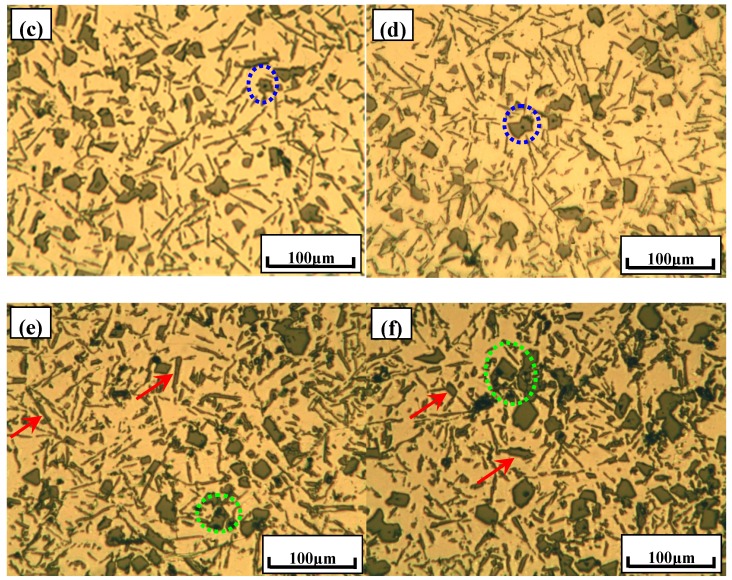
Typical microstructures of Al–18Si alloys with different melt treatments: (**a**,**b**) 0.2% Al–29Ca alloy; (**c**,**d**) 1.5% Al–6Y–2P master alloy; (**e**,**f**) 0.2% Al–29Ca; and, 1.5% Al–6Y–2P master alloys.

Figure 1 shows the typical microstructures of Al–18Si alloys with different melt treatments by using Al–29Ca and Al–6Y–2P master alloys. It is well known that the coarse primary Si with an average size even up to 100.0 μm could deteriorate the mechanical properties of Al–Si alloys and must be effectively refined. With the addition of 0.2% Al–29Ca master alloy, the primary Si can be modified to about 32.1 μm, but still presents irregular morphologies, such as polygon, as is illustrated in Figure 1a,b. Furthermore, it can be found that the eutectic Si were not efficiently modified and some coarse flaky eutectic Si were observed, as indicated by red arrows in Figure 1a,b. By comparison, the primary Si in Al–18Si alloys can be obviously refined with the addition of 1.5% Al–6Y–2P master alloy. The average size of primary Si has decreased to about 17.6 μm with the distribution being improved simultaneously. It was worth noting that there were some black particles that were embedded in primary Si act as the heterogeneous nuclei as circles shown in Figure 1c,d, which could be deduced to be AlP according to previous studies [23]. 

**Figure 2:**
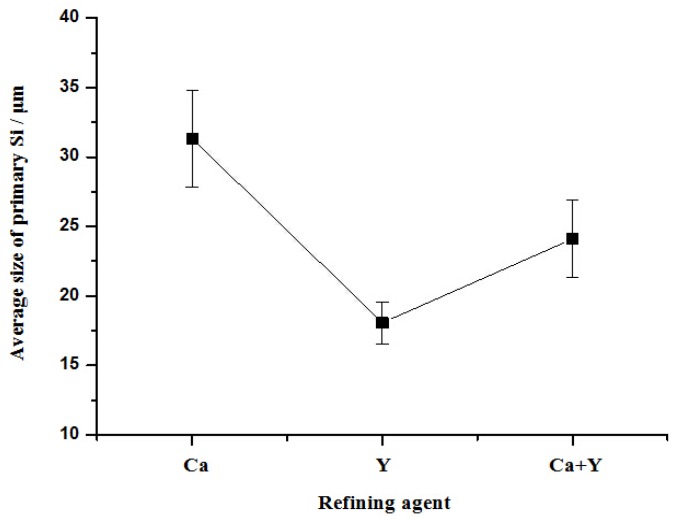
Variation of average size of primary Si in Al–18Si alloys with different melt treatments.

In order to investigate the combined effect of Ca and P on hypereutectic Al–18Si alloys, 0.2% Al–29Ca and 1.5% Al–6Y–2P master alloys were added into Al–18Si alloys and the typical microstructures of the alloys were illustrated in Figure 1e,f. It can be found that the average size of primary Si is about 24.1 μm with the standard deviation as 2.8 μm, which is smaller than that of alloy modified only by Ca, but relatively larger than that of alloy refined by P. Figure 2 shows the variation of average size of primary Si in Al–18Si alloys with different melt treatments. Furthermore, the coarse flaky eutectic Si was also found, which means that the eutectic Si cannot be effectively modified with the combination of Ca and P. In addition, there were some black compounds that were embedded in primary Si and/or in Al matrix, as indicated by circles in Figure 1e,f. According to the microstructural crack characteristic that was caused by the oxidization reaction, these particles can be deduced to be phosphides, which might contain a certain amount of calcium [28]. 

### 3.2. Phase Composition and Microstructure Analysis of Al–2Ca–3Y–1P Alloy

**Figure 3:**
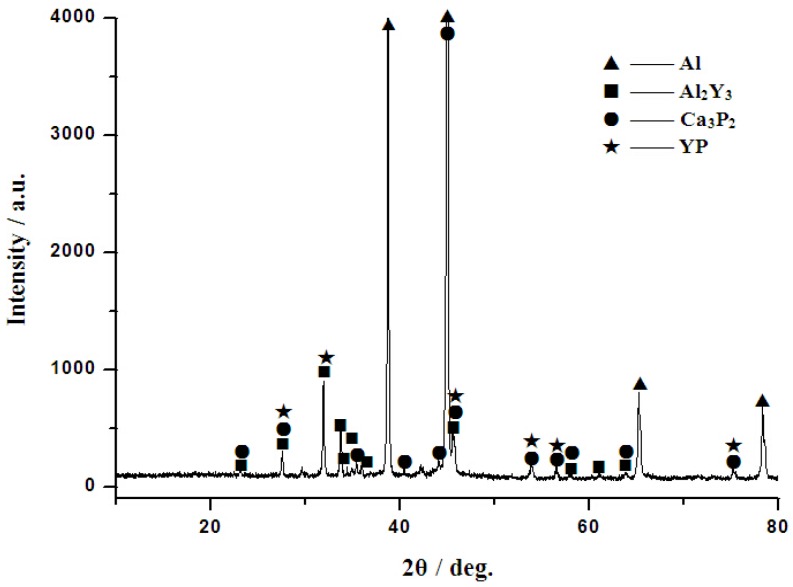
X-ray diffraction (XRD) patterns of Al–2Ca–3Y–1P alloy.

In order to investigate the existence form of Ca and its influence on the microstructure of aluminum alloy, Al–2Ca–3Y–1P alloy was prepared and the phase composition analysis of it was shown in Figure 3. It can be found that this alloy is composed of four phases, including α-Al, Al_2_Y_3_, Ca_3_P_2_, and YP. According to previous work [24], the novel Al–6Y–2P master alloy is composed of two phases, i.e., α-Al and YP. In this system, the atomic ratio of Y and P is about 1:1, indicating that all of the Y atoms would react with P to form YP without Y left. In comparison, from the phase compositions of these two alloys, it can be deduced that with the addition of Ca into Al–Y–P alloy, Ca would react with YP to form more stable Ca_3_P_2_ compounds, and residual Y would exist in the form of Al_2_Y_3_ and YP particles. 

**Figure 4:**
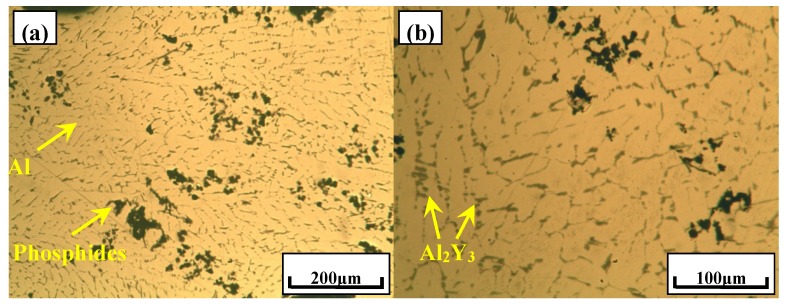
Typical microstructures of Al–2Ca–3Y–1P alloy. (**a**,**b**) Both of these two pictures are the typical microstructures of this alloy.

**Figure 5:**
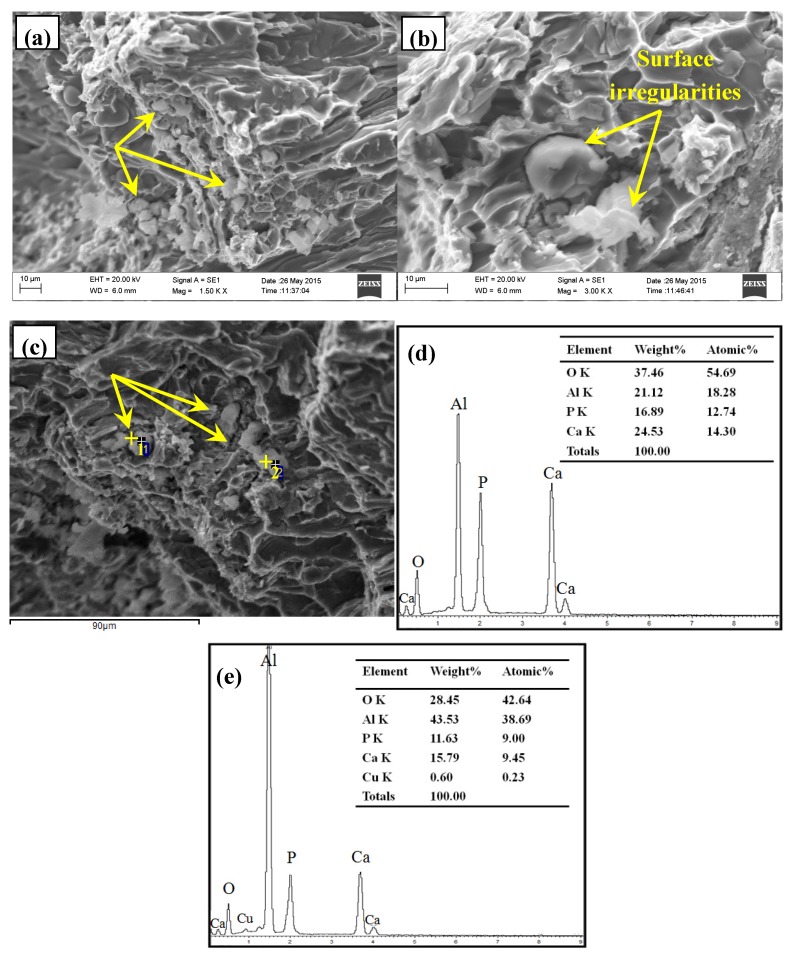
Field emission scanning electron microscope(FESEM) micrographs and energy dispersive X-ray spectrometer(EDS) analysis of Ca-rich compounds in Al–2Ca–3Y–1P alloys: (**a**–**c**) typical microstructures; (**d**,**e**) the corresponding EDS analysis for the spots 1 and 2 in (**c**), respectively.

**Figure 6:**
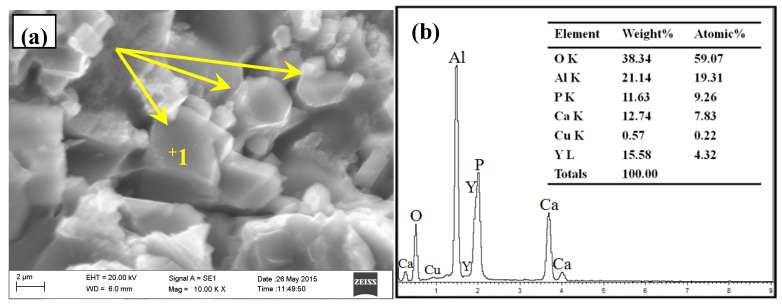
FESEM micrograph and EDS analysis of Y-rich phase in Al–2Ca–3Y–1P alloys: (**a**) microstructure; (**b**) EDS analysis for spot 1 in (**a**).

The typical microstructure of Al–2Ca–3Y–1P alloy was indicated in Figure 4. Besides of α-Al and Al_2_Y_3_ particles, as indicated in Figure 4, it can be seen that there were some black compounds in the matrix, which could be deduced to be phosphides due to their oxidation characteristics. Then, the fracture surface of Al–2Ca–3Y–1P alloy was investigated to obtain the morphology features in three-dimensional (3D) space. As shown in Figure 5a–c, there were a lot of particles that were embedded in the matrix, of which the surfaces are irregular, even some have wrinkles. According to the EDS analysis for the spots 1 and 2 in Figure 5c, it can be found that these particles were mainly composed of four elements, i.e., O, Al, P, and Ca. The enrichment of O and Al might be caused by the oxidization of phosphides and the aluminum matrix around these particles within the scope of EDS detection, respectively. The atomic ratios of Ca to P of these two particles were 1.12:1 and 1.05:1, respectively. When combined with XRD result in Figure 3, these particles can be proved to be Ca_3_P_2_ compounds. Besides of Ca_3_P_2_ particles with unsmooth surface mentioned above, some particles with regular geometrics were also detected, as indicated by arrows in Figure 6. Besides elements O, Al, and Cu, this compound contains 9.26 at.% P, 7.83 at.% Ca, and 4.32 at.% Y, which could be the evidence for the reaction process $2\mathrm{YP}+3\mathrm{Ca}\to {\mathrm{Ca}}_{3}P_{2}+2Y$.

### 3.3. Phase Compositionsand Microstructure Analysisesof Al–xSi–2Ca–3Y–1P Alloys (x = 6, 12, 18, 30)

**Figure 7:**
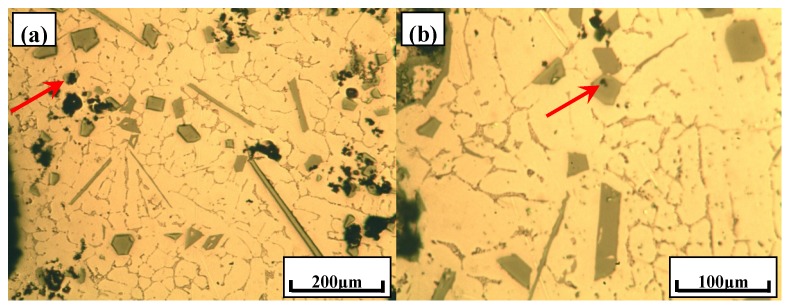

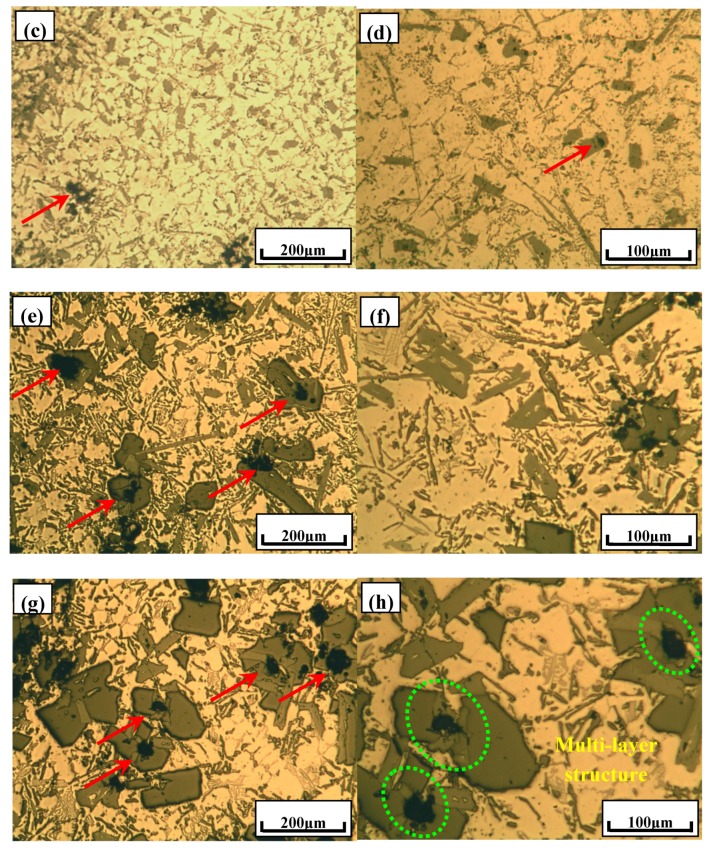
Typical microstructures of Al–xSi–2Ca–3Y–1P alloys: (**a**,**b**) Al–6Si–2Ca–3Y–1P; (**c**,**d**) Al–12Si–2Ca–3Y–1P; (**e**,**f**) Al–18Si–2Ca–3Y–1P and (**g**,**h**) Al–30Si–2Ca–3Y–1P.

Figure 7 shows the typical microstructures of Al–xSi–2Ca–3Y–1P (*x* = 6, 12, 18, 30) alloys. It can be observed that with the increase concentration of Si, the microstructures of Al–xSi–2Ca–3Y–1P alloys have remarkably changed. In the Al–6Si–2Ca–3Y–1P alloy, some compounds with the appearance of block and rod were found besides of α-Al dendrites, as illustrated in Figure 7a,b. Meanwhile, the black particles were also found in this alloy and some of them embedded in the blocky phase, as indicated by the arrows in Figure 7a,b. The microstructure of Al–12Si–2Ca–3Y–1P alloy was given in Figure 7c,d, from which it can be observed the blocky particles increase with the Si content. Furthermore, the black particles were also observed in Al–12Si–2Ca–3Y–1P alloy. With further increase of Si into Al–xSi–2Ca–3Y–1P alloys, these alloys possess the microstructural characteristics of hypereutectic Al–Si alloy. As clearly shown in Figure 7e,h, three phases were observed, i.e., α-Al, eutectic Si and primary Si. What is more, some multi-encapsulation structures were observed in these two alloys, as indicated by arrows in Figure 7e,h. Furthermore, it can be seen clearly that there were some black particles that were embedded in the center of the multi-encapsulation structures, which were encapsulated by some light grey particles. While, these light grey particles were surrounded by dark grey primary Si particles. In order to obtain the phase compositions and microstructure characteristics of these alloys in details, XRD analysises and FESEM observations were carried out. 

**Figure 8:**
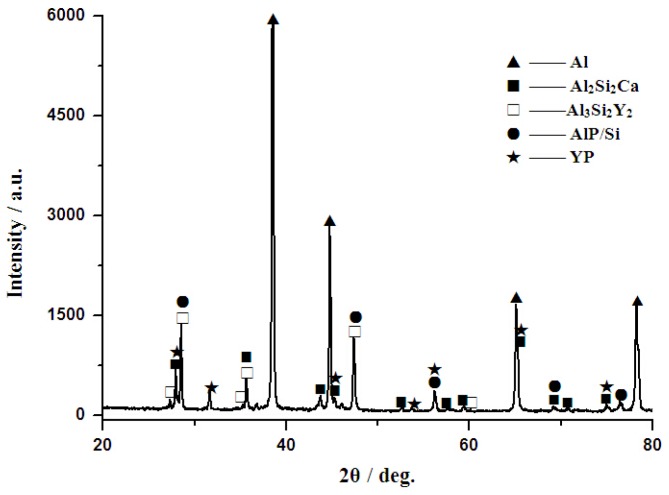
XRD patterns of Al–12Si–2Ca–3Y–1P alloy.

Figure 8 illustrates the XRD patterns of Al–12Si–2Ca–3Y–1P alloys. It can be found that this alloy is composed of six phases, i.e., α-Al, Al_2_Si_2_Ca, Al_3_Si_2_Y_2_, AlP, Si, and YP. According to the XRD patterns, the compound Ca_x_P_y_ was not detected. In comparison of phase compositions of Al–2Ca–3Y–1P and Al–12Si–2Ca–3Y–1P alloys, it can be obtained that Ca element would react with Si and Al to form Al_2_Si_2_Ca with the increase of Si content. As indicated in Figure 8, the diffraction lines exhibit peaks at 27.94°, 35.64°, 43.76°, 45.31°, and 65.30°, corresponding to the (011), (012), (110), (103), and (023) reflections of Al_2_Si_2_Ca with hexagonal structure (space group $P\bar{3}\;\mathrm{mL}$, *a* = 0.413 nm, *c* = 0.7145 nm), respectively. As is reported in reference [24], the Si atoms in the Al–Si melt could promote the structural evolution of YP to AlP accompanying with the release of Y, which may just be reason for the excellent composite modification performance of the developed Al–6Y–2P master alloy. In Al–12Si–2Ca–3Y–1P alloy, Al_3_Si_2_Y_2_ (space group C2/m, *a* = 1.022 nm, *b* = 0.4035 nm, *c* = 0.6617 nm, *β* = 101.36°) compounds were also synthesized based on XRD analysis. With the presence of Al_3_Si_2_Y_2_ phase in this alloy, it can be deduced that in this system Y element would react with Al and Si to form Al_3_Si_2_Y_2_ particles, and then residual Y would react with P to form YP particles. While, the excess P for the reaction between Y and P would combine with Al and precipitate in the form of AlP particles. 

**Figure 9:**
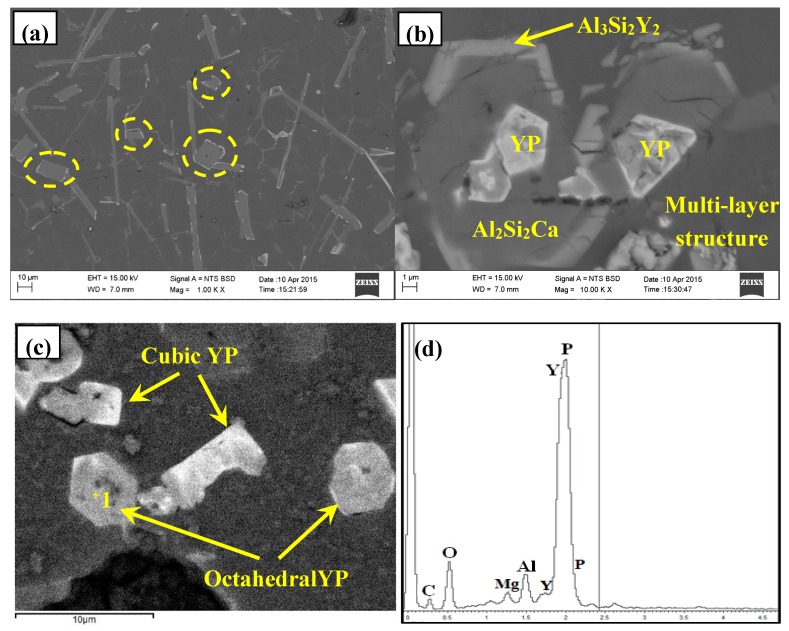
FESEM micrographs and EDS analysis of Al–12Si–2Ca–3Y–1P alloys: (**a**–**c**) typical microstructures; (**d**) EDS analysis for the spot 1 in (**c**).

FESEM observations of Al–12Si–2Ca–3Y–1P alloy were shown in Figure 9. It can be seen clearly that the blocy and needle shaped particles were encapsulated by a thin layer of light gray phase, as shown by circles and the partial enlarged drawing was given in Figure 9b. According to the EDS analysis, the particles embedded in the center of multi-encapsulation structure can be deduced to be YP compounds, which tend to exhibit cubic and/or octahedral morphologies due to its face-centred cubic structure [24].

**Figure 10:**
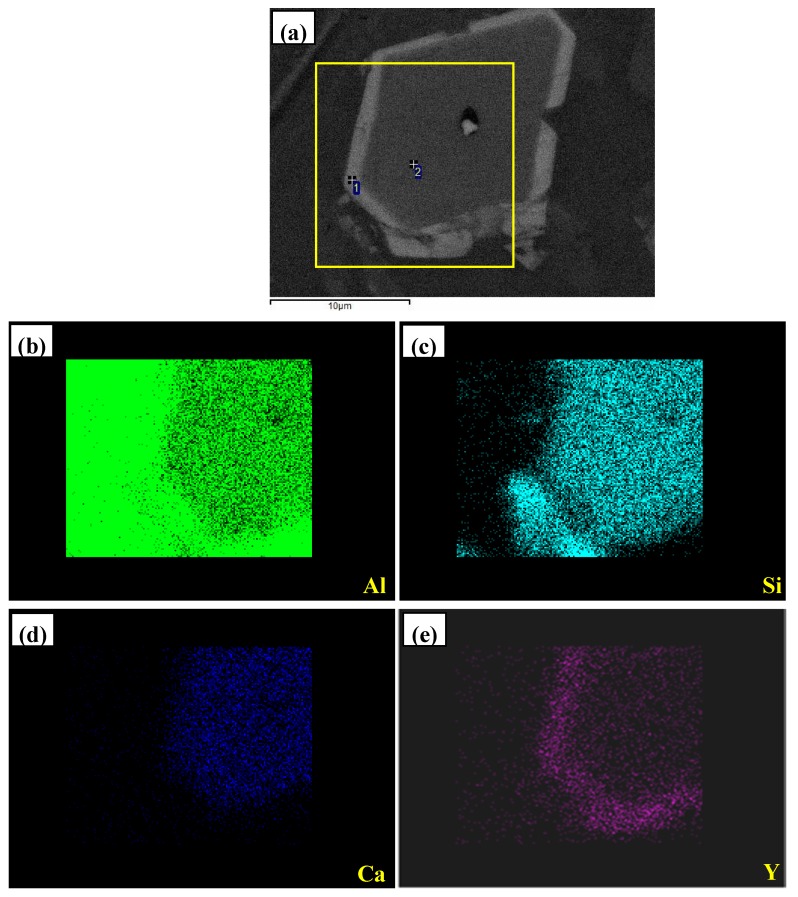
FESEM images of multi-encapsulation structure in Al–12Si–2Ca–3Y–1P alloys: (**a**) microstructures; (**b**–**e**) the X-ray images for elements Al, Si, Ca, Y, respectively.

By means of FESEM, the elemental analysis of multi-encapsulation structure was carried out and presented in Figure 10. It can be observed that the inner dark grey compound with the enrichment of Al, Si, and Ca elements was surrounded by a thin layer of light gray phase, which was composed of Al, Si, and Y elements. The corresponding quantitative analysises for the spots in Figure 10a were listed in Table 1. The spots 1 and 2 were mainly composed of 42.97 at.%Al, 30.17 at.% Si, 25.15 at.% Y and 29.33 at.% Al, 28.94 at.% Si, 13.92 at.% Ca, respectively. When combined with the XRD result, these compounds can be proved to be Al_3_Si_2_Y_2_ and Al_2_Si_2_Ca phase, respectively. Sun et al. [26] observed that the phosphorus was more likely to concentrate in the Al_2_Si_2_Ca phase, rather than in Ca_3_P_2_ or CaSi_2_, even with low content of Ca. Zaldívar-Cadena and Flore-Valdés [28] reported that calcium intermetallic particles exhibited needle-like and plate-like morphologies. Based on Figure 9 and Figure 10, it can be deduced that YP phase could be encapsulated by Al_2_Si_2_Ca and Al_2_Si_2_Ca could be surrounded by Al_3_Si_2_Y_2_. That is to say, the multi-encapsulation structure in Al–12Si–2Ca–3Y–1P alloy was composed of YP, Al_2_Si_2_Ca, and Al_3_Si_2_Y_2_ phases from inside to outside in order.

**Figure 11:**
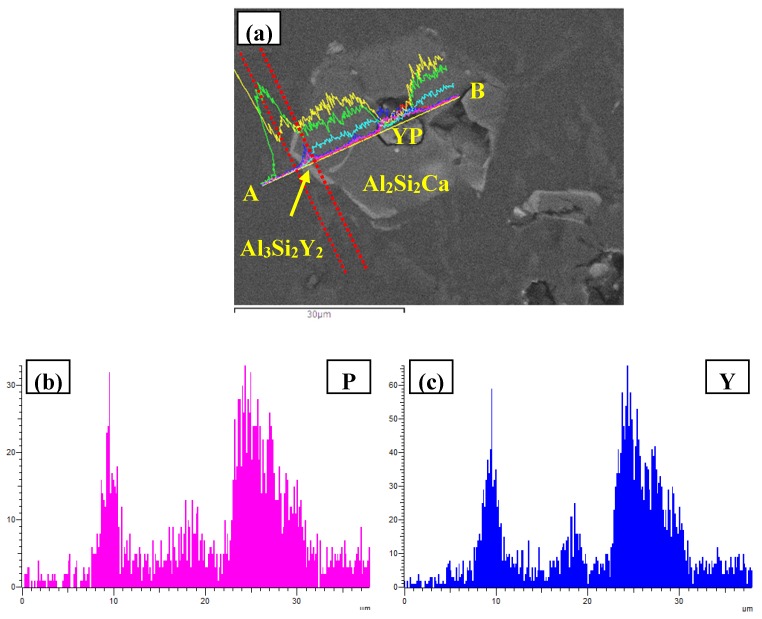

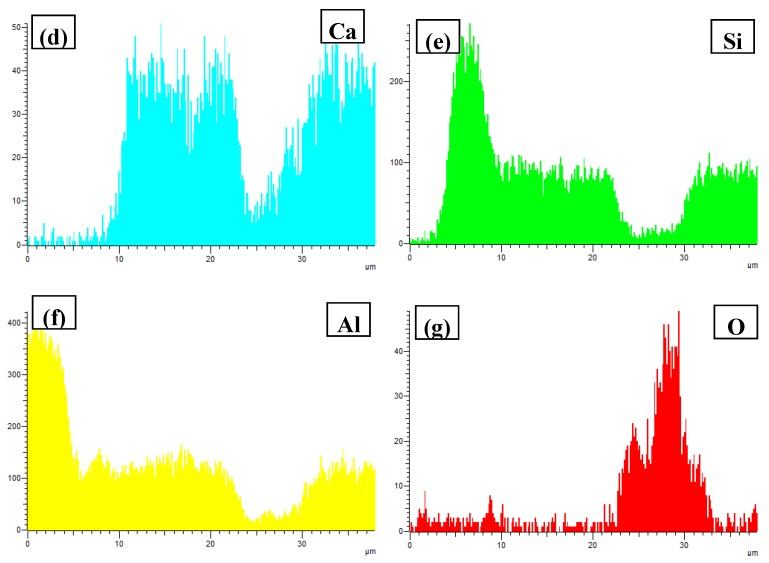
FESEM images of Al–12Si–2Ca–3Y–1P alloys: (**a**) microstructure; (**b**–**g**) EDS line scanning alalysises along A and B.

The FESEM micrographs and EDS line scanning alalysises along A and B were present in Figure 11. In the center of composite particle, a compound with the enrichment of Y, P, and Owas observed, and trace Al element was also detected. According to our previous work [24], the structural evolution of YP in Al–Si melt can take place as follows YP+Al→AlP+Y. Meanwhile, the enrichment of element O might be caused by the oxidation and hydrolysis of phosphides (YP and AlP) during the specimen preparation process. Based on this, the particles in the center part of multi-encapsulation structure can be deduced to be phosphides (YP and AlP). The hexagonal compound surrounded YP and AlP was proved to be Al_2_Si_2_Ca due to the enrichment of Al, Si, and Ca elements. In addition, the outmost thin phase that was highlighted by red dashed lines in Figure 11a was composed of Al, Si, P, and Y elements. Therefore, this compound can be deduced to be Al_3_Si_2_Y_2_, with solid solution of P element. As concluded above, the inner center of multi-encapsulation structure could be YP and/or AlP, which were encapsulated by Al_2_Si_2_Ca and Al_3_Si_2_Y_2_ from inside to outside in order. 

**Figure 12:**
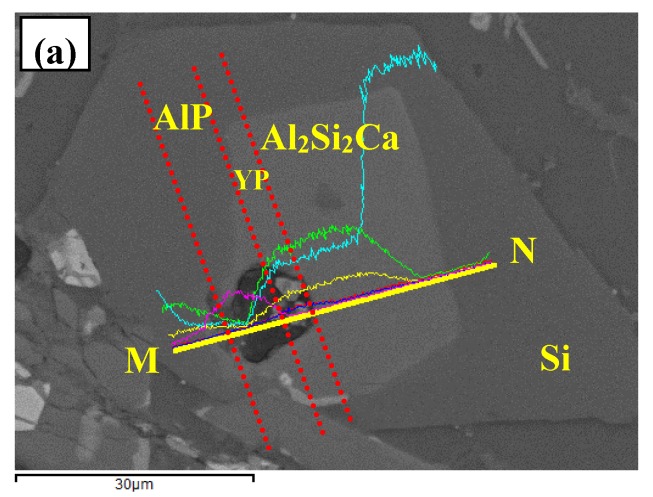

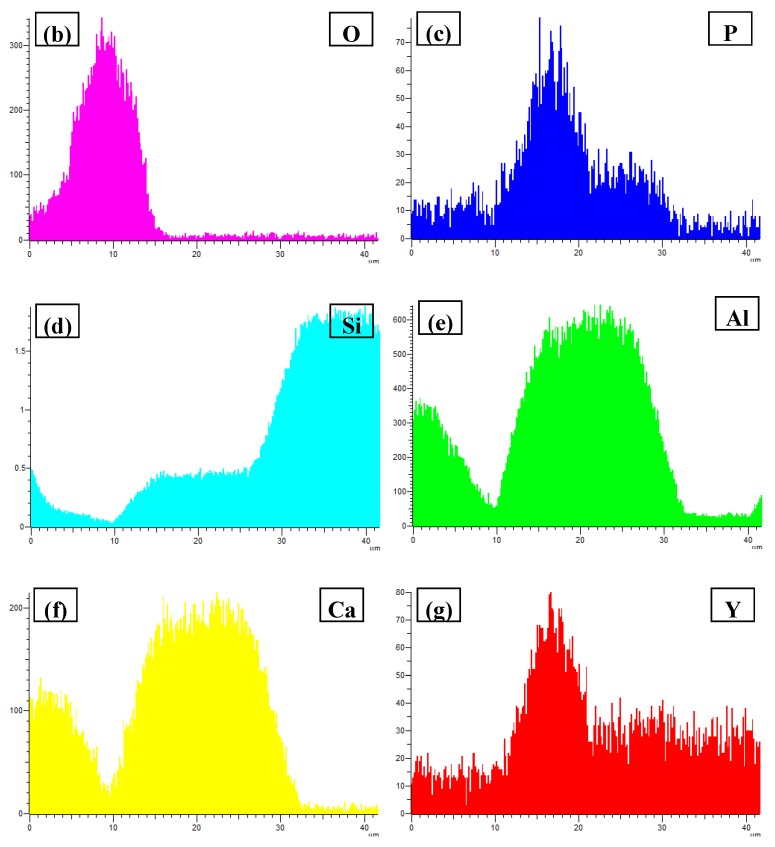
FESEM micrographs of Al–18Si–2Ca–3Y–1P alloys: (**a**) microstructure; (**b**–**g**) EDS line scanning alalysises along M and N.

With further increase of Si content, the multi-encapsulation structures in Al–18Si–2Ca–3Y–1P alloys were also observed, but slightly changed. As indicated in Figure 12, the particle in the core has oxidized severely, and elements Al, trace Y, and P were detected. While, the grey particle on the right side was observed to be enrichment of Y and P elements, as indicated by red dashed lines in Figure 12a. Therefore, the phase in the center of multi-encapsulation structure in Al–18Si–2Ca–3Y–1P alloy was phosphide (AlP and YP) and AlP particles were transformated by the structural evolution of YP in the melt. According to the chemical composition, the phase that surrounded the inner phosphides could be deduced to be the Al_2_Si_2_Ca phase. What is different for the multi-encapsulation structure in Al–18Si–2Ca–3Y–1P alloy, was that the outmost phase around Al_2_Si_2_Ca was primary Si, as clearly shown in Figure 12d. Therefore, the multi-encapsulation structure for this alloy was composed of phosphide (YP and/or AlP), Al_2_Si_2_Ca, and Si from inside to outside in order.

Based on the analysis above, it can be found that the novel Al–6Y–2P master alloy has a certain anti-poison resistence to impurity Ca for the modification of Al–Si alloys. Ludwig et al. [27] reported that Al_2_Si_2_Ca would precipitate even at a low Ca concentration of 39 ppm, and Ca additions exceeding 50 ppm have little additional benefit on the microstructure of Al–7Si alloy due to the formation of sable polyhedral Al_2_Si_2_Ca particle. It was observed that the main function of Ca was to reduce the number of P based nucleants for the eutectic Si by the formation of pre-eutectic Al_2_Si_2_Ca phase, which nucleates on AlP and by preferentially formation of the Ca_3_P_2_ phase [30]. It was worth noting that AlP phase can exist according to the analysis of multi-encapsulation structure, but some of them might be surrounded by Al_2_Si_2_Ca compounds. That is the reason for the limited refinement effect of Al–6Y–2P master alloy for Al–18Si alloys. In our investigation, 0.2% Al–29Ca (Ca: 580 ppm) and 1.5% Al–6Y–2P (P: 300 ppm) master alloys were added into the hypereutectic Al–18Si alloy, the primary Si was relatively refined due to the heterogeneous nucleation effect of AlP, but eutectic Si still remain the same, or even coarse, which was caused by the formation of Al_2_Si_2_Ca phase. 

In Al–xSi–2Ca–3Y–1P (*x* = 6, 12, 18, 30) alloys, two kinds of the multi-encapsulation structures were observed. The inner phase of these structures is phosphide, including AlP and YP, which were surrounded by hexagonal Al_2_Si_2_Ca phase. This may just be the reason for the restricted refinement effect of Al–6Y–2P master alloy with the presence of Ca in aluminum alloys. Furthermore, it can be found that the phosphides i.e., AlP and YP, would first precipitate during the solidification process, and then phosphides were surrounded by Al_2_Si_2_Ca compounds, which would be encapsulated by Al_3_Si_2_Y_2_ and/or primary Si phase later. With the presence of impurity Ca, the formation of encapsulation structures (YP–Al_2_Si_2_Ca and AlP–Al_2_Si_2_Ca) might be the reason for the weakening refinement effect of Al–6Y–2P master alloy on hypereutectic Al–Si alloys.

## 4. Conclusions

(1) With the presence of Ca and Al–6Y–2P master alloy, the primary Si was relatively refined to about 24.1 μm and eutectic Si still remain the same or even coarse, which indicates that novel Al–6Y–2P master alloy has a limited anti-poison effect of Ca, with an application in Al–Si alloys.
(2) In Al–2Ca–3Y–1P alloy, Al_2_Y_3_, Ca_3_P_2_, and YP compounds were detected besides of Al. Based on the microstructural analysis, it can be deduced that Ca could react with YP to form more stable Ca_3_P_2_ compounds, and residual Y would exist in the form of Al_2_Y_3_ and YP particles.
(3) In Al–xSi–Ca–Y–P (*x* = 6, 12, 18, 30)alloys, the multi-encapsulation structures were observed, which were composed of three layers, i.e., the phosphides (AlP and YP), hexagonal Al_2_Si_2_Ca, the Al_3_Si_2_Y_2_ or primary Si from inside to outside in order. The formation of encapsulation structure as YP–Al_2_Si_2_Ca and AlP–Al_2_Si_2_Camaynjust be the reason for the limited refinement effect of Al–6Y–2P master alloy for aluminium alloys.

## Figures and Tables

**Table 1:** The corresponding chemical compositions of spots in Figure 10a.

Spot	Element	Weight%	Atomic%
1	Al	26.89	42.97
	Si	19.66	30.17
	Ca	1.59	1.71
	Y	51.86	25.15
2	C K	13.38	27.81
	Al K	31.71	29.33
	Si K	32.56	28.94
	Ca K	22.35	13.92
